# Experiences of long-term benzodiazepine use and addiction amid changes in guidelines for the prescription of narcotic drugs: a qualitative study

**DOI:** 10.1186/s12889-025-23920-9

**Published:** 2025-08-05

**Authors:** Cecilia Krüger, Mathilde Hedlund Lindberg, Sofia Burmester, Johan Franck, Jeanette Westman

**Affiliations:** 1 Department of Neurobiology, Care Sciences and Society, Karolinska Institutet, Alfred Nobels allé 23, Huddinge, 141 52 Sweden; 2 Department of Medical Sciences, Clinical Pyschiatry, Uppsala University, Uppsala, 751 85 Sweden; 3 The Stockholm Centre for Dependency Disorders, Stockholm Health Care Services, Region Stockholm, Friskvårdsvägen 4, Stockholm, 112 81 Sweden; 4 Department of Clinical Neuroscience, Karolinska Institutet, Tomtebodavägen 18 A, 171 77 Stockholm, Sweden; 5 Academic Primary Care Centre, Region Stockholm, Solnavägen 1E, Stockholm, 113 65 Sweden

**Keywords:** Addiction, Benzodiazepines, Drug use, Patient experiences, Qualitative, Substance-related disorders

## Abstract

**Background:**

Addiction to sedative, hypnotic, and anxiolytic drugs (collectively referred to as benzodiazepines), medications commonly used to treat anxiety and sleeping problems, has been described as a hidden epidemic. Growing concerns about addiction and other negative outcomes associated with use of these narcotics have resulted in their regulation and recommendations for more restrictive prescription. However, there are few studies on experiences of long-term benzodiazepine use from the user perspective, and even fewer focusing on people with addiction, who may be the most affected by changing regulations. The aim of this study was to explore experiences of people with long-term use and addiction to benzodiazepines during a time of major changes in guidelines for the prescription of narcotic drugs.

**Methods:**

This qualitative study included nineteen individual interviews with adults (> 18 years) diagnosed with addiction to benzodiazepines who were undergoing treatment at an urban outpatient clinic in Sweden. The in-depth interviews were pseudonymized, transcribed verbatim, and analyzed using reflexive thematic analysis.

**Results:**

Two themes highlight the effect of changing prescription guidelines on long-term benzodiazepine users. The first theme, *benzodiazepines were not always seen as problematic*,* but neither were the risks discussed*, underscores the absence of psychiatric assessments and conversations about risks at the time of first prescription and highlights the need for clearer communication. The second theme, *entangled in continued benzodiazepine use and increasing restrictions on prescribing*, illustrates how addiction developed over time and describes how limited contact with prescribers facilitated long-term use, which often persisted until the participants were faced with deprescription.

**Conclusions:**

This study describes the experiences of individuals with long-term use and addiction to benzodiazepines during a time of shifting attitudes towards prescribing. The results highlight areas for improvement in care for this vulnerable group of patients, including the need for proactive and regular communication about risks of addiction and additional support during deprescription.

## Background

Addiction to sedative, hypnotic, and anxiolytic drugs is an ongoing global public health challenge that has received relatively little attention in the shadow of the opioid epidemic [1, 2]. This class of drugs includes benzodiazepines and related benzodiazepine-like hypnotics, which are frequently prescribed for the treatment of anxiety and sleeping problems, and are among the most widely used narcotics [3–5]. In this article, these drugs will be collectively referred to as benzodiazepines.

Although benzodiazepines offer symptom relief short-term, there is an ongoing debate about the benefits and risks, as they increase the risk of negative outcomes such as psychomotor impairment which can contribute to falls or traffic accidents, adverse cognitive effects, tolerance, addiction, overdose, and mortality; especially with long-term use [1–3, 6, 7]. The prevalence of long-term use—defined as use for six months or more within a year [6, 8]— is estimated to be 3% of the population in Sweden [8] and internationally [1–3, 9], and may be growing despite increasing awareness of the associated harms [4, 5]. For some individuals, long-term use of a low dose may not result in a clinical diagnosis of addiction, even if tolerance is common [4, 5]. However, an estimated 2% of people who use benzodiazepines are diagnosed with addiction, and rates are higher in vulnerable populations, including people with psychiatric illness, low socioeconomic status, use of other substances, and of older age [4, 10, 11].

When benzodiazepines were first introduced in the 1950s, they were generally marketed as being non-addictive, and these attitudes largely prevailed into the 1970s [4]. The first benzodiazepines were classified internationally as narcotics in 1971 [12]. Although prescribers began noting and studying adverse effects including symptoms of addiction in the 1980s [4, 5], regulation occurred gradually in different counties [5, 12, 13]. When benzodiazepine-like hypnotics were introduced to the market in the 1990s for the treatment of insomnia, they were initially thought to be a safer alternative and became widely used [4, 14]. However, in response to growing awareness of the risks over the past two decades, the whole class of drugs have become regulated under national drug control laws, made available by prescription only, and subject stricter clinical practice guidelines. In the first Swedish national treatment guidelines for anxiety and depression in 2010, benzodiazepines were given low priority, where use was acceptable in selected cases [15]. In 2011, all commonly prescribed drugs in this class were added to the list of narcotics by the Swedish Medical Products Agency (LVFS 2011:10) [10], and in 2017, the national treatment guidelines were updated to advise against the use of benzodiazepines [16]. Other evidence-based interventions, including psychological treatment and non-narcotic medications, are recommended instead of benzodiazepines for the treatment of anxiety and sleeping disorders [16, 17]. Although implementation of guidelines takes time, and uptake and perceptions on the restrictive recommendations by individual prescribers varies [5, 13], changes in guidelines directly impact prescribing norms and attitudes among health care providers [18]. In Sweden, the changes in guidelines have contributed to a decrease in the overall proportion of new users of benzodiazepines since 2015 [19], although little is known about patient experiences related to this trend of declining prescriptions.

Considering the widespread nature of benzodiazepine use, there is a dearth of research exploring the long-term experiences of these drugs from the user perspective. While several qualitative studies include benzodiazepine users [20], few delve into perceptions related to prolonged use and the impact of significant changes in regulation and prescribing of these medications over time. The perspectives of individuals with a history of long-term use are of interest as previously acceptable prescription patterns are now seen as contributing to addiction risk in updated guidelines. Even fewer studies investigate the experiences of people diagnosed with addiction specifically [21], the criteria for which include clinically significant impairment or distress related to their use (i.e., more use than intended, craving, continued use despite negative psychosocial or physical outcomes, tolerance, and withdrawal) [22]. The perspectives of long-term users formally assessed and diagnosed with addiction are also important, as they may have different experiences of changing prescriber attitudes compared to those without addiction.

The aim of this study was to explore experiences of people with long-term use and addiction to benzodiazepines during a time of major changes in guidelines for the prescription of narcotic drugs.

## Methods

### Study design and data collection procedures

This qualitative study was part of a larger project exploring patients’ experiences and perceptions of benzodiazepine use and addiction treatment that included 19 individual interviews completed in 2023 at a specialized outpatient addiction clinic in Stockholm [23]. Patients at the clinic can be referred by other health care providers or self-refer. Eligible participants were adults (≥ 18 years) with addiction to benzodiazepines, diagnosed according to Diagnostic and Statistical Manual of Mental Disorders, 5th edition (DSM-5) [22] criteria (i.e., sedative, hypnotic, and anxiolytic use disorder) who were in various stages of tapering treatment (i.e., gradual dose reduction) at the time of the interview. Non-Swedish speakers and individuals also in treatment for opioid use disorder were excluded.

Psychiatrists and nurses at the clinic identified eligible patients who were subsequently contacted by the researchers to assess interest in participation. Authors CK, JW, and a research nurse, none of whom were involved with treatment at the clinic, conducted the in-depth interviews. All participants provided written informed consent and answered a short questionnaire about their benzodiazepine use prior to the interview. Interviews followed a semi-structured guide, were audio-recorded, and lasted for an average of 57 min (SD = 20.4). Applying the concept of information power [24], we continuously assessed the transcripts for material richness during data collection and continued interviewing participants until we were satisfied that the sample held sufficiently rich data to provide meaningful insights into our research questions. During the preparation of this manuscript, we evaluated the qualitative checklist Standards for Reporting Qualitative Research [25]. The study was approved by the Swedish Ethical Review Authority (Dnr. 2019–05302).

The interview guide for the larger project included open-ended questions and prompts to explore experiences and perceptions of benzodiazepine use and addiction treatment and was previously published by the research group [23]. This study is a primary analysis of responses from all 19 participants to two overarching open-ended questions from the interview guide, which have not previously been analyzed or reported. These two questions were (1) “Tell me about your use of benzodiazepines,” which included several prompts about initiation and long-term use (i.e., how the person came to use benzodiazepines for the first time, the initial and later source(s), why they continued using benzodiazepines, associations with any symptoms, types of benzodiazepines used, and how using them affected their life) and (2) “Tell me about any positive effects or benefits you have experienced from using benzodiazepines.”

### Analytical approach

Interviews were pseudonymized and transcribed verbatim. Qualitative software (NVivo, version 14) was used to facilitate data organization and coding. Transcripts were analyzed using reflexive thematic analysis, which is described by Braun and Clarke as a qualitative approach that conceptualizes themes as patterns of shared meaning and embraces reflexivity and subjectivity as a resource in knowledge production [26, 27]. We approached the analysis inductively, highlighting the lived experiences of benzodiazepine use over time. Participants often used combinations of benzodiazepines and benzodiazepine-like hypnotics over extended periods; as such, substance-specific analyses were not conducted.

As first author, CK planned the analysis process for this study. CK and MHL independently read the Swedish transcripts, coding them in English before discussing interpretations and together creating a new set of codes that they developed into preliminary themes. CK and MHL identified illustrative quotations during the coding process and reviewed the preliminary themes to ensure meaningful coherence between codes, themes, and quotations. As a native speaker of English, CK translated the quotations from Swedish to English, and MHL conducted a back-translation. The authors modified quotations minimally and strove to preserve meaning and voice during translation, removing only sensitive information and including bracketed text as required to improve understanding. CK and MHL also assessed the transcripts for information on the initial source of benzodiazepines, reason for initiation, and psychosocial history, which are reported in Table 1.

All authors participated in reviewing the preliminary themes and organizing the manuscript text. The authors’ different interpretations of the data enriched the collaborative and reflexive rewriting process, which led to the final themes. The authors ascribe to a biopsychosocial model, which emphasizes how biological, genetic, psychological, sociocultural, and public health factors interact to influence the development of addiction [28]. CK has a background in public health and anthropology, SB in psychology and addiction care, JW in primary care and public health, and MHL and JF in psychiatry and addiction care, which added different perspectives to the analysis.

## Results

Participants included ten men and nine women, aged 25 to 75 years (mean 53.4, SD 14.6) (Table 1). 37% of the participants used only benzodiazepines, 21% only benzodiazepine-like hypnotics, and 42% a combination of both. Decade of initiation varied, with nearly half (42%) of the participants first using benzodiazepines prior to 2000. Most (74%) started to use benzodiazepines after receiving a prescription, while five (26%) first encountered them in other ways; however, all participants were prescribed benzodiazepines at some point during their use. Long-term use of both prescription and illicit benzodiazepines was common prior to receiving a diagnosis and entering addiction treatment. The mean duration of regular benzodiazepine use was 16.6 years (SD 13.7), with the shortest reported duration being 2 years.

In the interviews, participants reported that they started using benzodiazepines to treat symptoms of an anxiety disorder (58%), sleeping problems (16%), or a combination thereof (21%). Fifteen participants (79%) described experiences of psychosocial stress, trauma, or crises during their lifetime. Four (21%) said these experiences preceded prescription, five (26%) that they occurred after starting benzodiazepines, and six (32%) that they had happened both before and after starting benzodiazepines.

**Table 1:** Characteristics of population (*n* = 19) regarding benzodiazepine initiation, use and history of mental health problems

Characteristic		Mean (SD) or *N* (%)
**Age in years** ^a^		53.4 (14.6)
**Sex** ^a^	**Women** **Men**	9 (47%) 10 (53%)
**Decade of debut** ^a^	**1970–1979** **1980–1989** **1990–1999** **2000–2009** **2010–2019** **≥ 2020**	2 (11%) 3 (16%) 3 (16%) 3 (16%) 7 (37%) 1 (5%)
**Years with regular use** ^ab^		16.6 (13.7)
**Substance use prior to treatment** ^a^	**Benzodiazepines** *(alprazolam*,* oxazepam*,* diazepam*,* clonazepam)* **Benzodiazepine-like hypnotics** *(zopiclone*,* zolpidem)* **Both**	7 (37%) 4 (21%) 8 (42%)
**Initial source of benzodiazepines** ^c^	**Prescription**	**14 (74%)**
	Psychiatric care	3 (16%)
	Emergency department	1 (5%)
	Primary care	5 (26%)
	Prescription from health care (unspecified)	4 (21%)
	Physician outside of Sweden	1 (5%)
	**Non-prescription**	**5 (26%)**
	Took or received from parents	2 (11%)
	Received from friends	2 (11%)
	Purchased illegally online	1 (5%)
**Reason for initiation** ^c^	**Anxiety disorder**	**11 (58%)**
	Anxiety^d^	5 (26%)
	Panic disorder or panic attacks	6 (32%)
	**Sleeping problems**	**3 (16%)**
	**Anxiety**^**d**^ ** and sleeping problems**	**4 (21%)**
	**Suicidal ideation**	**1 (5%)**
**Psychosocial crisis, trauma, or stress**^c^	**Yes**	**15 (79%)**
	Prior to initiation	4 (21%)
	After initiation	5 (26%)
	Both	6 (32%)
	**No**	**4 (21%)**

^a^From questionnaire administered prior to interview.

^b^Regular use was defined as use at least 3 days a week

^c^Reported by the participant during the interview, assessed by CK and MHL. Classification of reason for initiation does not reflect a formal diagnosis. Instances of psychosocial crisis, trauma, or stress included examples such as sexual assault, intimate partner violence or abuse, and traumatic loss.

^d^Anxiety was self-reported, and type was not always specified by the participant.

The reflexive thematic analysis resulted in two themes, (1) *benzodiazepines were not always seen as problematic*,* but neither were the risks discussed* and (2) *entangled in continued benzodiazepine use and increasing restrictions on prescribing*, as well as subthemes that describe participants’ perspectives from initiation to deprescription (Table 2).

**Table 2:** Themes, subthemes, and representative quotations from study participants

Theme and subthemes	Quotation
***Theme 1. Benzodiazepines were not always seen as problematic, but neither were the risks discussed***	
Using benzodiazepines as a quick fix without discussing underlying mental health problems	*Well*,* what I didn’t know was that I had PTSD [post-traumatic stress disorder]*,* and I couldn’t sleep*,* I felt poorly which is why I got the sleeping pills*,* but it wasn’t that they told me*,* “you have PTSD.” (P16)*
Taking benzodiazepines like any other medicine, not understanding the risks	*I saw them just the same as [..] I mean I have other medications*,* heart medicines and for blood pressure and such*,* so it wasn’t any different than that*,* it was one [pill] every day. (P2)*
***Theme 2. Entangled in continued benzodiazepine use and increasing restrictions on prescribing***	
New ways of conceptualizing and relying on benzodiazepines developed over time	*It became a universal pill. (P10)*
Long-term use was facilitated by easy access to prescriptions	*I never had one of those talks*,* that*,* “we should maybe stop with these now and start something that isn’t addictive… sedative or*,* like*,* is less strong*,* or.. ” No*,* I just got them*,* without contact. Every month. (P12)*
Deprescription with insufficient support caused tension	*It was when I was forced to stop*,* I mean*,* we had like an acute crisis there*,* and then they took it [the prescription] away with the justification that I had become addicted. (P17)*

### Theme 1: Benzodiazepines were not always seen as problematic, but neither were the risks discussed

Participants described initial interactions with prescribers that were centered on pharmacologically resolving anxiety and sleeping problems, but included little discussion of underlying mental health status. When they first started using benzodiazepines, participants focused on the positive effects they experienced, such as increased function and a sense of normalcy. However, upon reflection, they felt insufficiently informed about potential risks at the start of use and indicated that more proactive communication would have been helpful. Retrospectively, participants viewed this as a breakdown in communication at the time of their first prescription. At the same time, they reflected that benzodiazepines were not always perceived as problematic when they first began using them—particularly among those who initiated use several decades ago. In those early stages, participants often regarded benzodiazepines as just another medication and did not fully grasp the risks associated with these narcotic drugs.

#### Using benzodiazepines as a quick fix without discussing underlying mental health problems

During the interviews, participants often mentioned a personal or family history of mental illness as a contributing factor to their initiation of benzodiazepine use.

It probably started with my being, a so-called “anxious child”, with anxiety that I couldn’t handle. I met, well, a psychologist before I started school. Had panic, death anxiety, and panic my whole childhood. (P11)

However, when reflecting on their initial encounters with prescribers, participants reported that discussions primarily focused on pharmacological symptom relief, and did not typically include assessment of potential underlying causes of their mental health difficulties. Participants said that they either refrained from disclosing or were not prompted to discuss histories of psychosocial stress, trauma, or crises at the time of their initial benzodiazepine prescription. Some participants were unaware of underlying psychiatric diagnoses when they first received the medication:

Well, what I didn’t know was that I had PTSD [post-traumatic stress disorder], and I couldn’t sleep, I felt poorly which is why I got the sleeping pills, but it wasn’t that they told me, “you have PTSD.” (P16).

Participants’ initial focus was often on the positive effects of benzodiazepines, including getting through a crisis, resolving symptoms, or regaining functionality in their daily lives. Those who started taking benzodiazepines without a prescription also explained their use as related to symptoms of undertreated mental illness, and later received prescriptions that allowed them to continue treating their symptoms: “It was for self-medicating purposes, then, that I acquired them [online], above all, perhaps, for a little social anxiety” (P13).

Participants said that they could not live a normal life or do *“*very basic things*”* (P9) without what they referred to as the good effect benzodiazepines had on anxiety, panic attacks, worry, and insomnia. Some participants even said that the symptoms they had experienced changed them, whereas with benzodiazepines, “I have, simply, been as I should be” (P3). They described taking a pill in the morning to “feel normal” (P7), “to be able to be out, like a normal person” (P14), and as necessary to facilitate the activities of daily life, including grocery shopping, commuting to school or work, or meeting new people.

It was very positive in the beginning, everyone thought so, because I was, like really, home for almost half a year, didn’t go past the [front] door. So everyone thought it was great that it worked. I mean, my family said so too. (P12)

#### Taking benzodiazepines like any other medicine, not understanding the risks

The combination of the positive effects they experienced and how easy it was to obtain a prescription—“[primary] health centers are quick to prescribe” (P6)—contributed to participants’ perception that the risks of using benzodiazepines were negligible. Participants said they were unaware of “how serious a drug this actually was” (P1). They felt that prescribers could have done more in early interactions to communicate the potential downsides of using these narcotic drugs. Several participants did not know about the addictive potential of benzodiazepines.

I mean, I was prescribed them and there was never anyone who said they were like, narcotic drugs or something, that it was a narcotic I was taking. (P5)

There were also misunderstandings about the expected duration of use. Some participants thought that treatment with benzodiazepines was intended to be lifelong, and that it was natural for their dose to increase over time. They were not aware of the risks of tolerance associated with this class of narcotics.

I saw them just the same as [...] I mean I have other medications, heart medicines and for blood pressure and such, so it wasn’t any different than that, it was one [pill] every day. (P2)

Upon reflection, participants wished for clearer, more proactive communication about the treatment plan and said that they had been unaware of the risks. They also shared their frustration about having been being prescribed narcotics as a first-line or common treatment.

I just think it is so freaking sick that doctors prescribe those kinds of medicines to people who are that young and also that– and such high doses too... like without control by the state or something. (P12)

After their own experiences with benzodiazepines, many participants supported restricting prescriptions, reasoning that the risk of addiction was too great to justify general use: “Not even in the case of a catastrophe, I think [...] I can’t see that there is any reason at all to prescribe this to anyone, actually” (P1).

### Theme 2: Entangled in continued benzodiazepine use and increasing restrictions on prescribing

Participants reported an average of nearly 17 years of regular benzodiazepine use (i.e., at least 3 days a week), often without consistent follow-up visits to health care settings where emerging signs of addiction might have noted. Many started with as-needed (*pro re nata* or PRN) prescriptions and initially thought they “did something good” (P11) by taking the drugs. However, signs of addiction developed over time as participants began relying on benzodiazepines in new ways and increasingly self-medicated. Participants described how maintaining prescriptions initially required minimal effort and continued uncontested for years, with little follow-up regarding changing use. However, the introduction of stricter guidelines increased tension in patient-provider interactions surrounding prescribing, particularly for people with long-term and high-dose use. Facing deprescription without sufficient support made some participants feel abandoned by the health care system.

#### New ways of conceptualizing and relying on benzodiazepines developed over time

As time passed, participants began using benzodiazepines more regularly and preventatively to treat their symptoms and manage various crises. They defined as-needed use themselves and began relying on benzodiazepines in a manner their prescriber may not have intended, using them for non-therapeutic reasons despite growing negative effects. Their conceptualization of the role of benzodiazepines in their lives also evolved. For instance, in recounting their path to addiction, several participants described viewing benzodiazepines as a lifeline:

It has been a lifeline to have the strength to go on, I mean, so I don’t have to put up with this anxiety and worry and that kind of thing. So it, well, that’s how it started. (P2)

Participants took additional doses to cope with challenges in daily life, self-medicated to neutralize strong feelings like melancholy, and used benzodiazepines to take a break from reality or stress:

... to just escape from here and, so to say, go someplace else and calm down, in order to then go back and deal with the things you have to deal with. (P5)

One participant described benzodiazepines as a “final protective embankment” (P4) which kept them from being overwhelmed by anxiety and provided a reliable means to ensure they could get enough sleep. Another participant used benzodiazepines to disassociate while staying in an abusive relationship, indicative of persistent use in a situation that could be mentally and physically dangerous:

I really felt, now you [partner] can’t get to me anymore, because I can... you can’t make me sad, you can’t make me scared or anything, I... it was like [...] it became my safety, I felt like, now I can turn off. (P10)

The new ways in which participants related to benzodiazepines over time were also apparent in the ways they ascribed the drugs with unexpected properties, such as improving their work by helping them “stress less and get more done” (P8) or making them “alert and clear-headed” (P18). As participants’ habits of self-medication increased, they started taking benzodiazepines to treat conditions including poor self-confidence, muscle tension, pain, low energy, fear, hunger, and headaches: “It became a universal pill” (P10). Participants’ changing conceptualizations of benzodiazepines and ways of relying on them were potential signs of developing addiction. However, according to participants, providers seldom asked about changing use patterns that could have warned of future problems, and participants themselves did not bring up changing self-medication practices with their prescribers.

#### Long-term use was facilitated by easy access to prescriptions

According to participants, the ease with which they could renew prescriptions facilitated their increased and prolonged benzodiazepine use. Participants who first used benzodiazepines in the 1980–1990s described meeting with what they referred to as an old guard of doctors who would ask them if their medication was working, and if they answered yes, would continue prescribing. As one participant said, “it has just rolled on, so... and then I have asked to get new, renewed prescriptions and I have just, like, gotten them” (P5).

Participants said follow-up visits were infrequent and that prescribers they had seen for many years neither asked probing questions about how they used benzodiazepines nor initiated conversations about risks of long-term use.

I just got my prescription every month, so I never had one of those talks, that, “we should maybe stop with these now and start something that isn’t addictive, sedative or, like, is less strong, or... ” No, I just got them, without contact. Every month. (P12)

Additionally, participants typically only contacted their prescriber when they needed a refill, a process that was often straightforward in their early years of use. Some participants described how previous providers had been “so very happy to prescribe medicine” (P15) or would agree to large renewals over the phone.

I could text them whenever, around the clock, and just say, “I’m out,” and they would prescribe 200 at a time, and sometimes they didn’t keep track, so sometimes I had 600 [benzodiazepines] at home. (P10)

Participants also gave examples of times when they felt prescribers minimized the risk of long-term use of a low dose or implied that the prescription was justified because of the serious nature of their situation. For example, one participant with previous benzodiazepine use who visited a new health care provider during a crisis recounted receiving a prescription with such a justification.

“In my 25 years, I have never prescribed such pills, but I am going to make an exception, and that exception is you.” (P12).

Participants felt that by agreeing to prescribe benzodiazepines or renew a prescription, prescribers validated their continued use. Over time, participants became more aware of the recurring problems in their lives that may have been caused or exacerbated by their long-term use of benzodiazepines. Despite growing awareness of the associated risks, many participants, including those who had tried alternative medications or psychological treatments, continued to view benzodiazepines as the only effective solution and chose to persist with their use.

It is a little bit of a rock and a hard place, like what is, yeah, what do you choose? You choose, well, to be able to live. (P9)

#### Deprescription with insufficient support caused tension

Participants reflected that obtaining a prescription became increasingly difficult over time and that interactions could become quite tense as providers’ attitudes surrounding prescription began to change: “there are a lot of doctors now who are very restrictive” (P4). Participants described increased external oversight of their prescribers, which they perceived as contributing to a more stigmatizing health care environment. At times, they felt judged by health care professionals for their benzodiazepine use or reported being labelled addicts when collecting their medications. Strategies to mitigate benzodiazepine risks—implemented by what participants referred to as younger doctors—often led to contention. Health care providers demanded to see drug lists before prescribing, refused to prescribe a participant’s preferred benzodiazepine, or issued progressively smaller prescriptions to encourage tapering.

I reached out to some doctor, but they were also a bit like that: “No...,” that they didn’t want to prescribe too much. It kind of started to be a bit more like that, that they became a little more... I mean, when I got the first [prescription], it seemed like they could prescribe however they wanted. (P7)

While increased exposure to more restrictive attitudes on benzodiazepine prescribing heightened participants’ awareness of risks, they often remained resistant to discontinuation. They feared losing access, especially when they still felt they needed benzodiazepines or did not see themselves as addicted. To avoid the risk of being denied a prescription by a new provider, participants often stayed with the same prescriber as long as possible. Yet, disagreements could occur even with long-term prescribers, particularly if they aimed to reduce or discontinue a prescription:

People who are addicted to these [benzodiazepines] can be very convincing to their doctors and very pushy too, and maybe it is hard then for the doctor to say no. (P1)

Participants’ responses to these changes in prescribing varied. They ranged from eventual acceptance that the type of prescribing they once knew might no longer be possible, to finding other ways to supplement their intake, to anger—especially when forced to discontinue against their will. Participants were especially upset when they felt their health care provider did not listen to them or understand the extent of their problems. Four months into addiction treatment, one participant still found it difficult to understand why they were denied a refill, and why emergency care would not give them a prescription when they felt they needed it:

I can get angry. Because if they walked in my shoes and had the anxiety that I have, well, then they would have prescribed them. (P4)

Participants expressed indignation at being abruptly forced to stop benzodiazepine use by providers who had long facilitated their treatment, and often described feeling abandoned by the health care system—particularly when faced with sudden deprescription or an inability to find a new prescriber.

It was when I was forced to stop, I mean, we had like an acute crisis there, and then they took it [the prescription] away with the justification that I had become addicted. (P17)

They recounted disappointment and frustration with the lack of support they felt from health care providers, as well as exhaustion from being shuffled between providers they described as inexperienced or unable to provide sufficient help, even when such help had been promised: “I was supposed to go to some relaxation... whatever it was... clinic, but I never got a time or anything [to go]” (P7). They also blamed health care providers for poor management of deprescription and suggested that more could have been done to support tapering or facilitate referral to addiction treatment.

... I think that they [primary health center prescribers] should know more about how to help patients taper out. But, I mean, it takes a lot of time, I guess, and maybe they don’t have time for that, but they could have referred me here [to the addiction clinic] then instead of searching by myself for several months. (P6)

## Discussion

This reflexive thematic analysis resulted in two themes: *benzodiazepines were not always seen as problematic*,* but neither were the risks discussed* and *entangled in continued benzodiazepine use and increasing restrictions on prescribing*. Participants’ journeys spanned decades during which benzodiazepine regulations evolved and guidelines for treating anxiety and sleeping problems shifted alongside attitudes towards prescribing narcotic medications. The study gives voice to individuals with long-term use of benzodiazepines who were subsequently diagnosed with addiction, illustrating the impact of stricter prescribing guidelines and highlighting areas for improvement in care.

The first theme, *benzodiazepines were not always seen as problematic*,* but neither were the risks discussed*, underscores evolving attitudes on benzodiazepines and the need for proactive communication about their risks, which could have helped address participants’ lack of information and the misinformation they had when they firstbegan using benzodiazepines. The absence of early discussions about risks may reflect changing attitudes towards these drugs rather than missed opportunities to communicate, especially since the majority of participants initiated use before the tightening of regulations in 2011. Concern about the addictive potential of benzodiazepines increased gradually among prescribers as knowledge of risks grew. This change, coupled with the emergence of other treatment options, led to shifts in prescribing practices over time. Reflecting on the impact of benzodiazepines on their lives, many participants supported the restrictive spirit of today’s guidelines for new users, even though this conflicted with their own experiences.

In addition to being an opportunity to highlight the risks of benzodiazepines, early prescribing occasions are an important window for psychiatric assessment. However, as described in subtheme one, participants seldom recalled receiving psychiatric evaluation or support for their mental health problems before their first benzodiazepine prescription. Based on the high number of individuals with history of psychosocial crisis, trauma, or stress in this population, assessment and treatment for these conditions may be particularly important, and its impact on individuals struggling with addiction to benzodiazepines is an area for further qualitative research. Current guidelines prioritize timely clinical assessment of mental health care needs [16] and recommend psychological treatment instead of or in addition to pharmacological treatments [7, 16, 17]. Diagnosing mental health problems and discussing non-narcotic treatment options in connection with benzodiazepine prescribing might help increase patients’ risk awareness and willingness to try alternatives.

Participants in this study described having positive feelings about benzodiazepines when they first started using them. They explained that the drugs reduced symptoms, made them feel normal, and helped them carry out daily activities. These results are in line with those of previous studies, which have found that people start using benzodiazepines to cope with psychological distress [21, 29, 30] or following failed self-care strategies [20]. Their positive view of benzodiazepines was reinforced by a belief that prescriptions were intended to continue indefinitely, as described in subtheme two. This pattern of thinking indicates inadequate risk awareness, as seen in previous qualitative studies [31–33], and underscores the need to discuss treatment aims with patients at the time of prescription. Prescribers in previous decades may have viewed benzodiazepines as suitable for long-term treatment and often did not raise concerns about associated risks during early consultations. In contrast, current prescribing guidelines encourage providers to clearly communicate the risks of benzodiazepines to patients, even when prescribed short-term. Ongoing patient education is also important for patients with long-term benzodiazepine prescriptions. Strengthening communication may help reduce prolonged use and the risk of addiction [7], a perspective supported by research suggesting that individuals concerned about adverse drug effects may be more receptive to behavioral therapies [34].

The second theme describes how participants became *entangled in continued benzodiazepine use and increasing restrictions on prescribing*, even as signs of addiction increased. This theme highlights the importance of regular follow-ups for individuals with long-term prescriptions, to avoid missing emerging signs and symptoms of addiction due to limited contact or insufficient inquiry into their use. The new ways people with long-term use came to rely on benzodiazepines and ascribed them with unexpected qualities, as described in subtheme one, are risks also observed in previous research [29, 32]. It is possible that some individuals chose to hide potentially problematic changes in their use and conceptualization of benzodiazepines from their prescriber due to concern about stigma or loss of access [35, 36]. However, subtheme two also illustrates that participants perceived the ease of prescription renewal as form of validation for their continued use, a finding supported by other studies [20, 21, 37]. Therefore, more frequent, patient-centered follow-ups could help health care professionals identify patients at risk and intercede earlier. Ahead of every renewal, it is important to assess and address potential inappropriate use, false beliefs about benzodiazepines, and signs of addiction.

Against the backdrop of the gradual adoption of new guidelines for prescribing benzodiazepines, it is important to emphasize that long-term users are particularly at risk—both of having received insufficient information in the past and of developing addiction due to prolonged use. Although addiction guidelines offer recommendations for tapering [38], this process requires prescribers to first assess and diagnose patients, and secondly, to have the necessary resources to support patients throughout tapering. The challenge of managing tensions around the prescribing and deprescribing of benzodiazepines has also been acknowledged by prescribers [18].

There is a risk that the introduction of guidelines may have unintended consequences [36], particularly for vulnerable patients, whose care and quality of life can be affected by unsupported or abrupt deprescription, regardless of whether they meet the criteria for addiction. Individuals with long-term prescriptions may also experience their diagnosis as sudden and stigmatizing, as the attitudes of their prescribers over the years could vary greatly. The tension in patient-provider relationships described subtheme three—especially when prescribers tried to get patients to taper or cease use against their will— demonstrates the need for enhanced frequency and clarity of communication about changing guidelines to reduce stigma, improve guideline adherence, and address misunderstandings and conflict [18, 20]. Although restrictions are essential for reducing addiction, abrupt cessation of benzodiazepines can be dangerous. Future guidelines could be strengthened by advising prescribers to proceed cautiously when modifying prescriptions for long-term users and by outlining clear steps to support safe deprescription. Enhancing health care professionals’ knowledge about addiction indicators, promoting patient-centered discussions about tapering, and encouraging referrals to addiction care are all important measures to prevent further harm among individuals with long-term use and addiction [20, 39].

### Strengths and limitations

Our collaborative discussions on the assumptions made during coding and while developing the preliminary themes was a strength of the analysis, as the reflexive process led to more nuanced final themes and increased trustworthiness. The in-depth exploration of patient perspectives was strengthened by the selected data extracts and quotations [26].

Perceptions of communication and experiences of changes to prescribing over time were the shared, organizing concepts in the themes [27] that resulted from our reflexive thematic analysis of open-ended questions about benzodiazepine initiation and long-term use. A potential limitation is that the interview guide did not include specific questions about patient-prescriber interactions or changes in prescribing over time. Moreover, given that participants’ recollections are retrospective, it is possible that their descriptions of misunderstandings at the time they first started using benzodiazepines were influenced by selective recall. It is also possible that they were unable to fully absorb information about risks while undergoing crises at the time of prescribing.

It should be noted that all study participants were in treatment for benzodiazepine addiction in the publicly financed health care system in Sweden at the time of their interview. The results may therefore be most transferable to populations undergoing treatment in a similar care context. The transferability of findings may also be limited by the lack of detailed demographic and comorbidity data, as well as specific information on patterns of benzodiazepine use (e.g., consistent vs. interrupted) and distinctions between prescribed and illicit sources. These factors, which were not collected in this study, may influence individual experiences.

## Conclusion

This qualitative study describes the experiences of individuals with long-term use and addiction to benzodiazepines. They reported receiving limited information on risks of use at initiation and insufficient support during long-term use and deprescription. The findings show how changes in regulations and clinical guidelines affect vulnerable patients and highlight areas for improvement in care.

## Data Availability

The interview datasets are not publicly available due the sensitivity of the material but may be made available by the corresponding author on reasonable request and subject to institutional approval.
